# Yawning Is More Contagious in Pregnant Than Nulliparous Women: Naturalistic and Experimental Evidence

**DOI:** 10.1007/s12110-021-09404-w

**Published:** 2021-07-13

**Authors:** Ivan Norscia, Lucia Agostini, Alessia Moroni, Marta Caselli, Margherita Micheletti-Cremasco, Concetta Vardé, Elisabetta Palagi

**Affiliations:** 1 Department of Life Sciences and Systems Biology, University of Torino, Torino, Italy; 2 Obstetrics and Gynecology Clinic, Pinerolo, Turin Italy; 3 Department of Biology, Unit of Ethology, University of Pisa, Pisa, Italy

**Keywords:** Empathy evolution, Emotional contagion, Prenatal attachment, Maternal–fetal attachment

## Abstract

Contrary to spontaneous yawning, which is widespread in vertebrates and probably evolutionary ancient, contagious yawning—yawning triggered by others’ yawns—is considered an evolutionarily recent phenomenon, found in species characterized by complex sociality. Whether the social asymmetry observed in the occurrence of contagious yawning is related to social and emotional attachment and may therefore reflect emotional contagion is a subject of debate. In this study we assessed whether yawn contagion was enhanced in pregnant women, a cohort of subjects who develop prenatal emotional attachment in preparation for parental care, via hormonal and neurobiological changes. We predicted that if yawn contagion underlies social and emotional attachment, pregnant women would be more likely to contagiously yawn than nonpregnant, nulliparous women of reproductive age. We gathered data in two different settings. In the experimental setting, 49 women were exposed to video stimuli of newborns either yawning or moving their mouth (control) and we video-recorded the women during repeated trials to measure their yawning response. In the naturalistic setting, 131 women were observed in a social environment and their yawning response was recorded. We tested the factors influencing the yawning response, including the reproductive status (pregnant vs. not pregnant). In both settings, yawn contagion occurred significantly more in pregnant than nonpregnant women. By showing that pregnant women were most likely to respond to others’ yawns, our results support the hypothesis that the social variation observed in yawn contagion may be influenced by emotional attachment and that yawning in highly social species might have been coopted for emotional contagion during evolution.

Whereas spontaneous yawning is independent from the perception of others’ yawns, contagious yawning occurs when the yawn emitted by a subject (trigger) acts as a releasing stimulus (sensu Tinbergen and Perdeck 1951) and elicits yawning in another subject (responder) (Provine 1989). Although morphological variants are present in yawns, especially in primates (e.g., chimpanzees, *Pan troglodytes,* Vick and Paukner 2010; geladas, *Theropithecus gelada,* Palagi et al. 2009; Tonkean macaques, *Macaca tonkeana,* and Japanese macaque, *M. fuscata,* Zannella et al. 2017; humans, *Homo sapiens,* Provine 1986, 2012), spontaneous yawning is probably a plesiomorphic (ancestral) trait because it has been recorded in a wide array of vertebrates (Baenninger 1987). To the contrary, contagious yawning between conspecifics has been observed thus far in a relatively small number of species (Palagi et al. 2020) and may be an apomorphic trait, which appeared more recently in vertebrate evolution. With one exception (*Pongo pygmaeus*; van Berlo et al. 2020), the species exhibiting yawn contagion between conspecifics usually live in highly social groups: namely, all the extant hominine species (chimpanzees: Anderson et al. 2004; Campbell and Cox 2019; Campbell and de Waal 2011; bonobo, *Pan paniscus*: Demuru and Palagi 2012; Tan et al. 2017; but see Amici et al. 2014 on a very small sample size; humans: Provine 1986, 1989), two species of cercopithecines (geladas and Tonkean macaques; Palagi et al. 2009; Palagi and Norscia 2019), nonprimate mammals (lions, *Panthera leo*: Casetta et al. 2021; wolves, *Canis lupus lupus*: Romero et al. 2014; sheep, *Ovis aries*: Yonezawa et al. 2017; elephant seals, *Mirounga leonina*: Wojczulanis-Jakubas et al. 2019; domestic pigs, *Sus scrofa*: Norscia et al. 2021), and one social bird species (budgerigar, *Melopsittacus undulates*: Gallup et al. 2015).

One of the most remarkable aspects of intra-specific yawn contagion is that it shows social asymmetry in all the species where this aspect has been investigated (Campbell and de Waal 2011, 2014; Demuru and Palagi 2012; Massen et al. 2012; Norscia and Palagi 2011; Palagi et al. 2009; Romero et al. 2014). The yawning response is most likely or precisely triggered by yawns coming from individuals that are “socially relevant” to the potential responders, even though the communicative value of the triggering yawns (e.g., threat, tiredness) can vary. For example, in humans the yawning response is highest between familiar subjects (Norscia and Palagi 2011). In chimpanzees, living in social groups characterized by male dominance, males seem to respond more when the triggering yawn comes from the dominant males (Massen et al. 2012), whereas in bonobos, living in groups with female dominance, females seem to be more effective in eliciting others’ yawns (Demuru and Palagi 2012). Indeed, bonobos and chimpanzees preferentially attend familiar subjects of the dominant sex (Lewis et al. 2021). In geladas, the female dyads—which are responsible for maintaining group cohesion—showed the most precise matching of different yawning types (Palagi et al. 2009).

The social attachment between individuals seems also to affect the rates of yawn contagion. In dogs (*Canis lupus familiaris*), the evidence of interspecific yawn contagion (dog/human) and its modulation is mixed (for review: Neilands et al. 2020; Palagi and Cordoni 2020); in wolves, top rates of intraspecific yawn contagion were found between strongly bonded subjects (with bonding being measured by assessing the level of affinitive behavior; Romero et al. 2014). Adult chimpanzees (but not immature chimpanzees, Madsen and Persson 2013) yawn more in response to the yawns of ingroup than outgroup members (Campbell and de Waal 2011). Bonobos (in vivo but not when exposed to video stimuli; cf. Tan et al. 2017) show the highest yawning response between closely bonded individuals (Demuru and Palagi 2012; Palagi et al. 2014). A similar situation occurs in geladas, with yawn contagion being greatest between individuals that affiliate the most (Palagi et al. 2009). In humans, yawn contagion is higher in kin and friends than in acquaintances and strangers (Norscia and Palagi 2011; Norscia et al. 2016), and the familiarity bias remains when the yawns are heard but not seen (Norscia et al. 2020).

Based on neuroethological evidence, it has been hypothesized that in highly social species yawning may have been coopted during evolution for emotional contagion, a basic building block of empathy (de Waal and Preston 2017; Palagi et al. 2020). However, at present, it is highly debated for both human and nonhuman animals whether the social asymmetry observed in yawn contagion depends on interindividual bonding, possibly reflecting emotional attachment—as postulated by the Emotional Bias Hypothesis (EBH)—and/or on other factors, such as attentional levels, social dominance, or as-yet undefined aspects of the social setting (Adriaense et al. 2020; Kapitány and Nielsen 2017; Massen and Gallup 2017; Palagi et al. 2020).

Emotional contagion and empathic processes are assumed to have evolved from mother–offspring bond (for review: Preston 2013). Pregnant women are particularly suitable to investigate the link between yawn contagion and bonding because they undergo heavy psychological, physiological, and neurobiological changes leading to the development of maternal attachment and caregiving (Barba-Müller et al. 2019; Napso et al. 2018; Tichelman et al. 2019). These changes often alter body systems so that pregnant women perform and act differently (e.g., with respect to dietary choice, motor activity, sensitivity to emotional stimuli) than nonpregnant women in the general population (Crozier et al. 2009; Gradmark et al. 2011; Moya et al. 2014; Osório et al. 2018).

Although proposing different underlying mechanisms, definitions, and measures, (Brandon et al. 2009), the psychological literature addressing attachment theory (originally introduced for the postpartum period; Bowlby 1969) converges in indicating that mother-infant bonding starts long before birth, during pregnancy (Ferrari et al. 2016; Sadeghi and Mazaheri 2007; Salehi and Kohan 2017; Sedgmen et al. 2006). During gestation, women develop what Rubin (1975:149) called a sense of “we-ness,” later defined as prenatal attachment, the emotional and psychological bond between the mother and her unborn child (Brandon et al. 2009; Rossen et al. 2017). The mother-infant bonding quality developed in pregnancy is important because it is positively associated with the mother-infant bonding quality after birth (Tichelman et al. 2019).

Psychobiological changes during pregnancy, involving hormonal and maternal brain adaptations, occur in both human and nonhuman mammalian females to support the transition to parenthood (Kim 2016; Lonstein et al. 2015). In women, the establishment of prenatal attachment is sustained by recent neurobiological evidence. Via magnetic resonance imaging (MRI), Hoekzema et al., (2017) found that during pregnancy women’s brains undergo dramatic, long-lasting changes in areas that significantly overlap with areas involved in the Theory of Mind (ToM) (i.e., anterior and posterior cortical midline and specific sections of the bilateral lateral prefrontal and temporal cortex; Hoekzema et al. 2017). ToM, among other aspects, is related to the ability to read others’ emotions (affective ToM; Abu-Akel and Shamay-Tsoory 2011). Brain changes are also linked to the development of maternal attachment and can significantly predict the quality of future mother-infant attachment (Hoekzema et al. 2017).

Psychological and neurobiological changes are interconnected with the massive hormonal variations that occur in women during gestation (Barba-Müller et al. 2019; Glynn and Sandman 2011). Changes in the so-called maternal brain (including areas especially involved in maternal caregiving) are mediated by glucocorticoids, prolactin, and oxytocin, whose levels increase across pregnancy (Kim and Strathearn 2016; Napso et al. 2018; Prevost et al. 2014; Slattery and Hillerer 2016). Moreover, prolactin–Growth Hormone (GH) family and neuroactive hormones, including melatonin and its precursor serotonin, prepare pregnant women to adequately care for their offspring by impacting on different physiological functions (Lévy 2016; Napso et al. 2018). Oxytocin is the neuroactive hormone that is thought to play a major role in the development of maternal attachment and, more generally, social bonding in humans and other animals (Decety et al. 2016). Although contextual and interindividual factors can mitigate or even reverse the effects of oxytocin (Beery 2015; Olff et al. 2013), during pregnancy oxytocin is involved in the emergence of mother-infant emotional bonding and, in humans, also in the mental representations typical of such bonding (Decety et al. 2016; Feldman et al. 2007).

In summary, yawn contagion may be related to emotional attachment (as predicted by EBH), and pregnant women represent a cohort of subjects that is biologically and psychologically “equipped” for mother-infant emotional attachment (Barba-Müller et al. 2019; Brandon et al. 2009; Palagi et al. 2020; Tichelman et al. 2019). Hence, to check for further evidence of the association between yawn contagion and social attachment, possibly reflecting emotional attachment (de Waal and Preston 2017), we focused on the yawning response in pregnant women. In particular, we predicted that if social asymmetry in contagious yawning is also driven by interindividual attachment—a proxy of emotional attachment—contagious yawning would occur at higher rates in pregnant compared to nulliparous women.

## Material and Methods

The data for this study were collected from two distinct categories of women: pregnant women and nulliparous women—that is, women who were not pregnant and had no children. For the purpose of this study, we excluded from the nulliparous category women who had previously been pregnant because such experience is known to alter the maternal brain and the perception/recognition of infant and adult facial expressions (Hoekzema et al. 2017; Kim 2016; Matsunaga et al. 2018).

Data were gathered in two different settings: the experimental setting, with the study subjects being isolated and exposed to video stimuli under controlled conditions (via trials), and the naturalistic setting, with the observational data collected on the study subjects in their environmental social context (no trials involved). The study subjects were different for the two data collection types (experimental and naturalistic). On one hand, the experimental approach allowed the control or removal of certain variables (age, bond) but subjects were extrapolated from their social context. On the other hand, the naturalistic approach allowed the verification of the possible influence of pregnancy on the yawning response in ecological (but also more variable) conditions. Because either setting has advantages and drawbacks, we combined the experimental and naturalistic approach.

A yawn response can be considered to occur within 5 min after perceiving someone else’s yawn (the trigger’s yawn) (Provine 1986), with a peak in the first minute (Palagi et al. 2014; Provine 2005). However, in the fourth minute there is a higher probability of autocorrelation (meaning that the presence of a yawn performed by a subject at t_0_ increases the probability to have another yawn by the same subject at *t*_(0+X)_, where X is the increasing unit of time; Kapitány and Nielsen 2017). Therefore, we considered only responses that occurred within a three-minute time slot from the yawn emitted by the trigger (on video in the experimental condition and live in the naturalistic setting), in line with several previous works and to facilitate comparison (Anderson et al. 2004; Demuru and Palagi 2012; Norscia and Palagi 2011; Norscia et al. 2016, 2020; Palagi et al. 2014).

### Experimental Setting

**Fig. 1:**
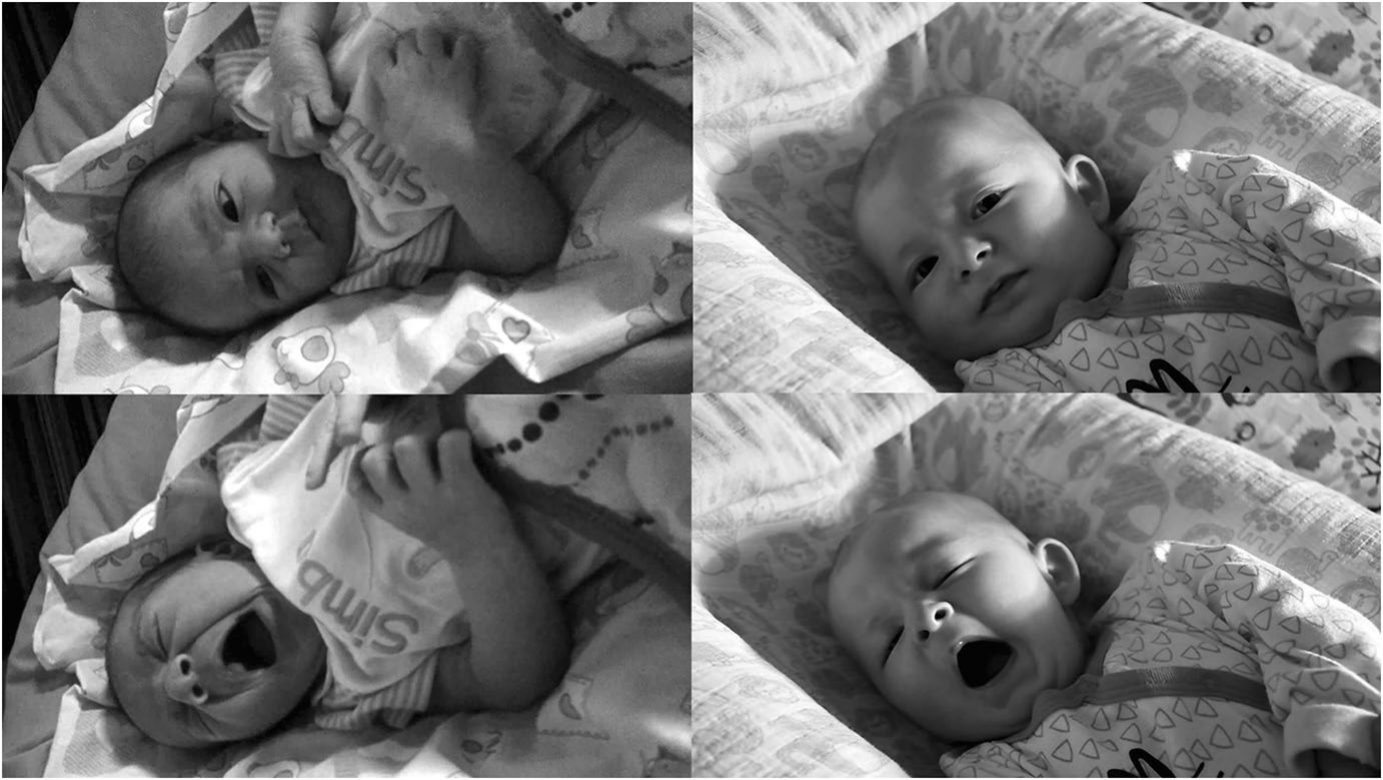
Screenshots from the experimental video showing the control condition (top: babies moving their mouths) and the yawning condition (bottom)

The video used for the experimental procedure (detailed in the next section) was composed of a black-and-white stimulus video and black-and-white neutral landscape videos. The stimulus video was built by joining 4–8 s clips showing two newborns (respectively 3 days and 3 months old, within the full breastfeeding period) while yawning (experimental condition) or moving their mouth (control condition) (Fig. 1). The experimental and control clips of each newborn had the same duration and were extracted from the same videos within seconds, so they had the same framing, context, luminosity, contrast, and background. The total duration of the stimulus video (either yawning or mouth movements), including both babies, was 45 s. The clips were provided by the newborns’ parents. Both parents signed a release document granting free use of the clips, including the possibility of showing and manipulating them for this research.

The videos with neutral landscapes were built from clips downloaded from a specialized website (pixabay.com). A beep sound added at the beginning of the video was downloaded (as.wav file) from freesound.org. Both the videos and the beep are available under Creative Commons CC0 license (Public Domain Dedication). Under this license, video and sound uploaders have waived their copyright and related or neighboring rights to the videos, which can be freely adapted and used without attributing the original author or source.

The full video (.avi) was obtained by merging the different videos into a single video (1820 × 720 px) in the following order: 20 s of neutral landscape video, the first stimulus video (45 s), 5 min of neutral landscape video, the second stimulus video (45 s), 3 min of neutral landscape video. The central period of 5 min of neutral landscape ensured that 3 + 2 min elapsed from the first to the second round of stimuli, in order to reduce the probability of autocorrelation.

The whole video was converted into black and white to remove any possible reference to the baby’s gender (based on color of clothing or other cues). The video editing was carried out via the freeware Avidemux 2.7. Two versions of the full video were assembled, and both videos showed yawning and control stimuli; one video showed the yawning stimuli before the control (YC video), and the other showed the control stimuli before the yawn (CY video).

### Experimental Setting and Study Subjects

A total of 292 experimental trials were carried out from June 2018 to January 2020, between 9:00 am and 7:00 pm. The trials with pregnant women were carried out at Dr. C. Vardé’s Obstetrics and Gynecology Clinic (Pinerolo, Italy). Pregnant women (*N* = 26, age range: 24–43 years old) participated in the trials on a voluntary basis during their monthly checkups at the clinic. Pregnancies ranged from 105 to 277 days (delayed delivery). Depending on their availability, the women underwent 1–6 trials (*M* = 2.29; *SD* = 1.21). Nulliparous women of reproductive age (*N* = 23, age range: 24–40 years old) were tested at the Department of Life Sciences and Systems Biology (University of Turin, Italy) or in private houses. The sample size, with variably repeated measures, allows the use of Generalized Linear Mixed Models.

In all cases the trials were carried out in an isolated room to avoid any distraction or interference. The sample only included women who had slept at least 5 h, had no certified or declared disorders, and were not under pharmacological treatments that could alter the yawning rates (e.g., involving the use of psychoactive substances). The subjects were white Italians, as inferred from physical traits and their last names. The newborns were unknown to all the tested subjects.

In compliance with the applicable regulations (Italian Legislative Decree no. 196/2003; EU General Data Protection Regulation 2016/679), women signed an informed consent in which they agreed to participate in the trials and granted permission (1) to be video-recorded during the experiment and (2) to have the video used for the purposes of this study. The exact purpose of the trials (recording yawning during pregnancy) was not revealed until the end of the study period when the women were told that the experiment was about the evaluation of attentional levels during pregnancy.

For each woman the trials were carried out by same experimenter (LA, AM, or MC). Right before the trial began, the woman was accompanied from the waiting room to a separate room and invited to sit on a chair in front of the screen, located at the height of the woman’s eyes. A camera had been previously located behind the screen at about 1.20 m distance, above the screen, so the face of the woman could be entirely recorded on video. The operator pressed the start button and the video started, preceded by 1-s blue screen with a beep sound. 20 s of neutral landscape clips were shown, while the operator left the room. After the first 20 s of neutral landscapes, when the woman was alone in the room, the first stimuli video started, marking the actual beginning of the trial. The woman watched the entire video, including stimuli and neutral sequences, lasting 9.21 min. The stimulus sequence (yawning/control or control/yawning) was randomized both within and across subjects (the neutral landscape clips were the same).

The face of the woman was recorded during the entire duration of the trial via a Canon Legria HFR36 to measure the number of yawns she exhibited while watching the experimental and the control video and in the following three minutes. The trials were carried out using a 15″ screen laptop (Core Processor i3–i5, 2.40–3.7 GHz, 64 Bit, 4–8 GB RAM). The videos shown to the women occupied the entire screen.

*Video analyses and data collection.* Data were entered anonymously, by assigning an alphanumeric code to each woman. Videos were analyzed via the free software VLC 3.0.6 (©VideoLAN). For each trial the following pieces of information were included in the dataset: time, woman’s code, reproductive status (nulliparous or pregnant), age, whether the woman yawned or not in the three minutes following the display of the first yawning or control stimulus, condition (yawning/control), the seconds the woman spent looking at the screen (attentional level), stimulus presentation sequence (yawning/control or control/yawning).

We categorized a yawn as such in these cases: (a) jaws open in a wide gape, deep inhalation, eye closing or narrowing (open yawns); (b) lip sealing, deep inhalation and at least one of the following patterns: nostril opening, eye narrowing, vacuum swallowing (nose yawns) (Provine 2012; for vacuum swallowing: present study).

Data were extrapolated from the videos independently by MC based on the above categorization and recoded by IN and EP. The average Cohen’s for yawn recognition was κ = 0.94, and only yawns with 100% agreement were included in the analysis.

The levels of attention to the stimuli were overall excellent (*M*_yawning_ = 44.952 s; *SD* = 0.271; *M*_control_ = 44.794 s; *SD* = 0.978; *M*_pregnant_ = 44.864 S; *SD* = 0.733; *M*_nulliparous_ = 44.855 s; *SD* = 0.842).

### Naturalistic Setting: Study Subjects and Data Collection

For the data collection in the naturalistic setting we also considered pregnant and nulliparous women (as defined above). Observational data on the pregnant women (*n* = 81) were collected live (with no video) in the waiting rooms of the Department of Obstetrics and Gynecology of the Hospital of Pinerolo (Turin, Italy) and data on the nulliparous women (who were not pregnant and had no children; *N* = 49) of reproductive age (in their twenties, thirties, or early forties) were collected at the Department of Life Sciences and Systems Biology (students during breaks, before and after classes) and in other settings (e.g., workplaces, social events) in 2019. These data also included observations of two pregnant women. Data were collected when conditions allowed unobstructed observation of all the individuals present, focusing on small, isolated groups, in absence of external perturbing events (e.g., strong noises, sudden interruptions by others entering the room). During data collection the identity of each subject was anonymously indicated via an alphanumeric code. The women included in the database were observed for at least 30 min and did not show repeated or abnormal displacement behavior.

All of the nulliparous women were known by at least one of the authors who collected the data (IN, EP, LA), their basic information was known, including their reproductive state. As a further confirmation, none of the nulliparous woman showed signs of pregnancy or delivered between the end of data collection and the time this article was written. The pregnant women were not known personally, but the basic information needed for this study was obtained through conversation with the data collector (LA). Data were collected from between around 9:00 am and 11:00 pm by using the all-occurrences sampling method (Altmann 1974), with the women not knowing that they were being under observation and without any evident external source of disturbance. Notes were taken—unnoticed—on the mobile phone or on paper. The training on yawn identification was carried out by IN and EP on the videos collected from June to December 2018 in the experimental setting. Only open yawns were considered in the naturalistic setting.

When a subject yawned spontaneously (no faked yawn) in presence of at least one observer (potential responder), the following data were entered in the calculation sheet: time, yawner dummy coded identity (trigger), the dummy coded identity of the woman (potential responder) who could perceive the yawn (distance within 5 m), reproductive status of the potential responder (whether the woman was pregnant or nulliparous), social bond between trigger and potential responder (stranger or acquaintances), whether the woman yawned in the three minutes after the trigger’s yawn (yawning response). We collected 308 yawning bouts in the presence of pregnant and/or nulliparous women.

Based on Norscia and Palagi (2011), the social bond was defined as follows: strangers = subjects who met for the first time; acquaintances = subjects who personally knew each other and whose relationship was based on a third external element—that is, work/university (colleagues), friends in common (friends of friends), patient-doctor relationship. Only the cases in which the bond was known to at least one of the authors were considered.

As explained above, we considered the yawning response as occurring within a three-minute time slot from the yawn emitted by the trigger. To reduce the possible autocorrelation effect during yawn trains (subsequent yawns occurring within 3 min following a triggering yawn), only the first yawn following the last trigger’s yawn was recorded as response.

### Statistical Elaboration

To check for possible differences in the two cohorts of women (pregnant and nulliparous), we ran the parametric *t*-test for two independent samples on age (normal distribution: Kolmogorov–Smirnov test, *p* = ns) and nonparametric Mann–Whitney tests for two independent samples (Siegel and Castellan 1988) on experimental time and declared sleep hours (nonnormal distribution, Kolmogorov–Smirnov test, *p* < 0.05). Montecarlo randomization (10,000 permutations) was applied for experimental time and sleep hours to account for pseudoreplication (same women repeated in different trials).

To analyze the data from the experimental trials we ran three GLMMs (Generalized Linear Mixed Model). A GLMM was run to verify what contextual factors could have an effect on the presence of yawning response (*N* = 292 cases). The occurrence of a yawning response was entered as a dependent, binomial variable (coded as presence = 1, absence = 0). The following fixed factors were included in the full model: condition (factor variable: yawning video stimulus = 1; control video stimulus = 0), attention level (numeric variable: number of seconds the woman looked toward the video stimuli), video sequence (factorial: yawning/control = 1, control/yawning = 2), time period (factorial variable coded as follows: 09:01 am–12:30 pm = 1; 12:30–16:00 pm = 2; 16:00–19:30 pm = 3) (Giganti and Zilli 2011). The woman’s dummy coded identity (potential responder) was entered as random factor.

Two additional GLMMs were ran to check which individual factors could influence the yawning response for either the yawning (*N* = 146) or the control (*N* = 146) condition. In both models, the occurrence of yawning response was entered as a dependent, binomial variable (coded as presence = 1, absence = 0). The fixed factors in the full model were age (numeric variable) and reproductive status (factorial variable: nulliparous = 0; pregnant = 1). The woman’s dummy coded identity (potential responder) was entered as random factor.

A GLMM was also run to verify what factors could have an effect on the presence of yawning response in naturalistic conditions (*N* = 308 cases). The occurrence of yawning response was entered as a dependent, binomial variable (coded as presence = 1, absence = 0). The fixed factors in the full model were (a) reproductive status (factorial variable: nulliparous = 0; pregnant = 1), (b) social bond linking trigger and potential responder (factorial variable: strangers = 0; acquaintances = 1), and (c) time period (factorial variable coded as follow: 1 = 09:00 am–12:30 pm; 2 = 12:30–16:00 pm; 3 = 16:00–19:30 pm; 4 = 19:30–23:00 pm; Giganti and Zilli 2011). The variables bond and reproductive status were inversely correlated (Kendall’s Tau-b =  − 0.794, *p* < 0.05) so they were included in the model as possibly having a divergent influence on the yawning response. The dummy coded identities of trigger and potential responder were entered as random factors owing to the variably repeated or unrepeated measures on the subjects.

We fitted the models in R (R Core Team 2020; version 3.5.3) using the function lmer of the R-package lme4 (Bates et al. 2015). We verified the significance of the full model in comparison to a null model that only included the random factors (Forstmeier and Schielzeth 2011). We used a likelihood ratio test (Dobson 2002) to test this significance (ANOVA with argument “Chisq”). We calculated the *p* values for the individual predictors based on likelihood ratio tests between the full and the null model using the R-function “drop1” (Barr et al. 2013). Since the response variable was binary, we used a binomial error distribution (link function: logit).

## Results

### Experimental Setting

No significant difference was found between the two cohorts with respect to age distribution (*t*-test for independent samples, *N*_nulliparous_ = 23; *N*_pregnant_ = 26; *t* = 1.728; df = 47; *p* = 0.091), experiment time (Mann–Whitney via Montecarlo randomization: *N*_nulliparous_ = 69; *N*_pregnant_ = 77; *U* = 2531.00; *p* = 0.617), and declared sleep hours (Mann–Whitney via Montecarlo randomization: *N*_nulliparous_ = 69; *N*_pregnant_ = 77; *U* = 2274.00; *p* = 0.123).

**Fig. 2:**
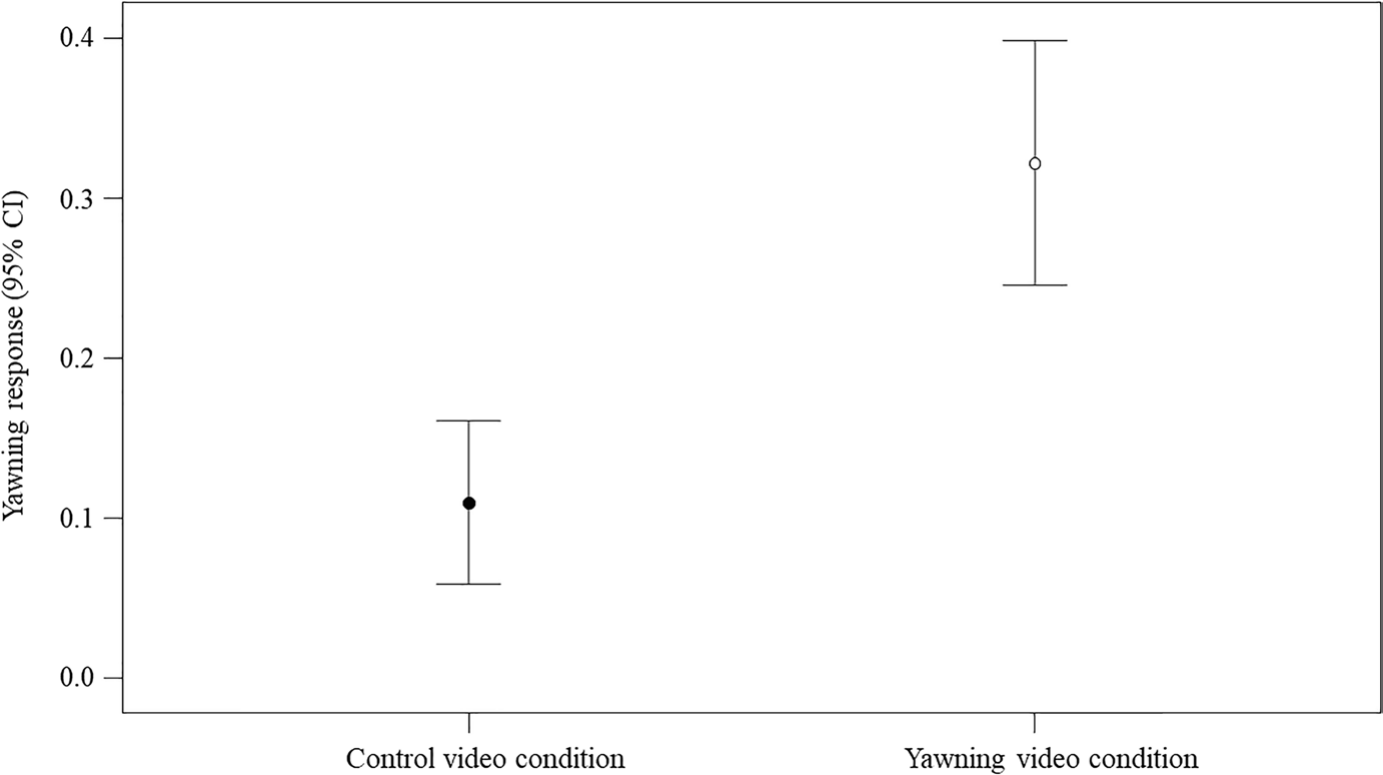
Effect of the type of the video condition (yawning/control) on the yawning response (experimental setting). Line plot showing the yawning response (Y axis) in the experimental setting as a function of the condition (yawning/control; X axis). The presence of a yawning response was significantly more likely in the yawning (*M* = 0.3322; *SE* = 0.039) than in the control (*M* = 0.120; *SE* = 0.026) condition (statistical results: Table 1), which confirms the presence of yawn contagion. Mean (circle) and 95% confidence interval (bars) are indicated

We ran a GLMM to check for the possible influence of contextual factors (condition: yawning video stimulus/control video stimulus, attention level, time slot and video sequence) on the yawning response. We found a significant difference between the full model fitted versus the null model (likelihood ratio test: χ^2^ = 34.997, df = 5, *p* < 0.001). Therefore, we moved on with a drop1 procedure. The GLMM indicated a significant effect of the condition (Table 1), with the yawning response being higher in the yawning than in the control video condition (Fig. 2). No significant main effect was found for the other variables.

**Table 1:** Results of the GLMM including the following fixed factors: condition (factor variable: yawning video stimulus = 1; control video stimulus = 0), attention level (numeric variable: number of seconds the woman looked toward the video stimuli), video sequence (factor variable: YC = 1, CY = 2), time period (factor variable: 09:00 am–12:30 pm = 1; 12:30–16:00 pm = 2; 16:00–19:30 pm = 3). The identity of the potential responders (Responder) was included as random factor

	Estimate	*SE*	*z*	*p*
(Intercept)^a^	− 16.953	20.673	—^a^	—^a^
Condition (Y)^b,c^	2.275	0.489	4.655	< .001
Attention	0.305	0.459	0.664	.507
Time period (2)^b,c^	− 0.976	0.631	− 1.546	.122
Time period (3)^b,c^	− 0.770	0.669	− 1.151	.250
Sequence (2)^b,c^	0.195	0.410	0.474	.635

Full vs. null model: χ^2^ = 34.997, *df* = 5, *p* < .001

^a^ Not shown as not having a meaningful interpretation

^b^ Estimate ± SE refer to the difference of the response between the reported level of this categorical predictor and the reference category of the same predictor

^c^ These predictors were dummy coded, with Condition (C), Time period (1), Sequence (1) being the reference categories

**Fig. 3:**
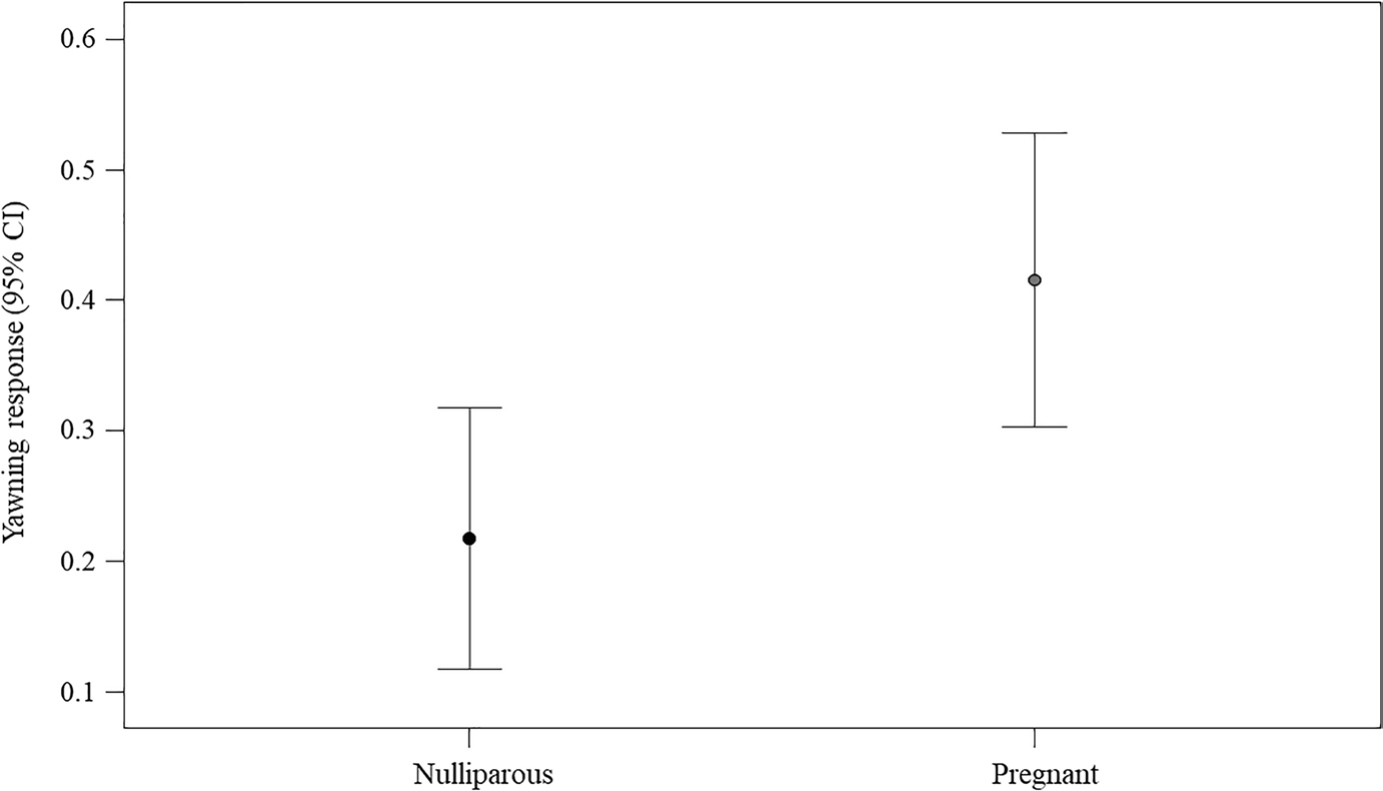
Effect of the reproductive status on the yawning response (experimental setting). Line plot showing the yawning response (*Y* axis) in the experimental setting as a function of the reproductive status of the woman potential responder (nulliparous/pregnant; *X* axis). The presence of a yawning response in the yawning video condition was significantly more likely (*M* = 0.416; *SE* = 0.057) in pregnant than in nulliparous (*M* = 0.217; *SE* = 0.050) women (Statistical results: Table 2). Mean (circle) and 95% confidence interval (bars) are indicated

Subsequently, we ran two GLMMs on either yawning video condition data or control video condition data to test the possible effect of two individual factors (age; reproductive status: pregnant/nulliparous) on the yawning response. For the control video condition model, we found no difference between the full and the null models (likelihood ratio test: χ^2^ = 0.391, df = 2, *p* = 0.822), with no predictor having a significant main effect on the response variable (age, *p* = 0.653; reproductive status, *p* = 0.596). Regarding the yawning video condition model, we found a significant difference between the full and the null models (likelihood ratio test: χ^2^ = 6.140, df = 2, *p* = 0.046). Only reproductive status had a significant main effect on the response variable (Table 2), with pregnant women being more likely to respond to another’s yawns than nulliparous women (Fig. 3).

**Table 2:** Results of the GLMM including the following fixed factors: age (numeric variable) and reproductive status (factor variable: nulliparous = 0; pregnant = 1); the identity of the potential responder was included as random factors

	Estimate	*SE*	*Z*	*p*
(Intercept)^a^	2.746	2.742	—^a^	—^a^
Reproductive status(1)^b,c^	1.684	0.813	2.071	.038
age	− 0.165	0.096	− 1.715	.086

Full vs. null model: χ^2^ = 6.140, df = 2, *p* = .046

^*a*^ Not shown as not having a meaningful interpretation

^*b*^ Estimate ± SE refer to the difference of the response between the reported level of this categorical predictor and the reference category of the same predictor

^*c*^ This predictor was dummy coded with Reproductive status (0) being the reference category

### Naturalistic Setting

We ran a GLMM to check for the possible influence of different factors (reproductive status: pregnant/nulliparous; social bond: strangers/acquaintances; time period) on the yawning response.

**Fig. 4:**
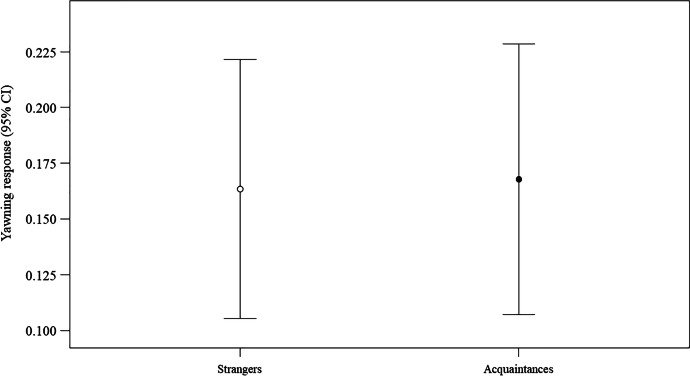
Effect of the social bond on the yawning response (naturalistic setting). Line plot showing the yawning response (*Y* axis) in the naturalistic setting as a function of social bond between trigger and potential responder (strangers/acquaintances; *X* axis). The presence of a yawning response was significantly more likely between acquaintances (Mean ± SE: 0.168 ± 0.031) than between strangers (*M* = 0.164; *SE* = 0.029) (statistical results: Table 3). Mean (circle) and 95% confidence interval (bars) are indicated

**Fig. 5:**
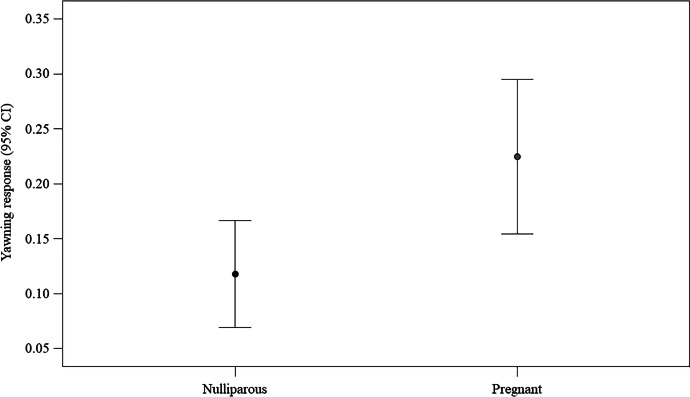
Effect of the reproductive status on the yawning response (naturalistic setting). Line plot showing the yawning response (Y axis) in the naturalistic setting as a function of the reproductive status of the woman potential responder (nulliparous/pregnant; X axis). The presence of a yawning response was significantly more likely in pregnant (*M* = 0.225; *SE* = 0.036) than in nulliparous (*M* = 0.118; *SE* = 0.025) women (statistical results: Table 3). Mean (circle) and 95% confidence interval (bars) are indicated

We found a significant difference between the full model fitted versus the null model (likelihood ratio test: χ^2^ = 11.183, df = 5, *p* = 0.048). Therefore, we moved on with a drop1 procedure. The GLMM indicated a significant effect of bond and reproductive status (Table 3), with the yawning response being higher in acquaintances than strangers (Fig. 4) and in pregnant more than nulliparous women (Fig. 5). No significant main effect was found for the time period.

**Table 3:** Results of the GLMM including the following fixed factors: reproductive status (factorial variable: nulliparous = 0; pregnant = 1), social bond linking trigger and potential responder (factorial variable: strangers = 0; acquaintances = 1), time period (factorial variable coded as follow: 09:00am–12:30 pm = 1; 12:30–16:00 pm = 2; 16:00–19:30 pm = 3; 19:30–23:00 pm = 4). The coded identity of trigger and potential responder were entered as random factors

	Estimate	*SE*	*z*	*p*
(Intercept)^a^	− 7.115	2.152	—^a^	—^a^
Reproductive status (1)^b,c^	4.650	1.745	2.664	.008
Bond (1)^b,c^	4.150	1.792	2.315	.021
Time period (2)^b,c^	1.009	0.869	1.161	.246
Time period (3)^b,c^	0.719	1.244	0.578	.563
Time period (4)^b,c^	− 1.572	1.342	−1.172	.241

Full vs. null model: χ^2^ = 11.183, df = 5, *p* = .048)

^a^ Not shown as not having a meaningful interpretation

^b^ Estimate ± SE refer to the difference of the response between the reported level of this categorical predictor and the reference category of the same predictor

^c^ These predictors were dummy coded, with Reproductive status (0), Bond (0), and Time period (1) being the reference categories

## Discussion

The results from both the experimental and the naturalistic data converge in indicating that women’s reproductive status had an effect on contagious yawning, which was more likely to occur in pregnant than in nulliparous women (here defined as women who were not pregnant and had no children). As a matter of fact, pregnant women were more likely to respond than nulliparous women to both video yawns of unknown infants in the experimental trials and live yawns from adults in the naturalistic setting (Tables 2 and 3; Figs. 3 and 5). This finding, presented for the first time with this study, provides support to the Emotional Bias Hypothesis (EBH) because yawn contagion was highest in the category of women characterized by enhanced social attachment predisposition, owing to the biological and psychological changes typical of the gestation period (Barba-Müller et al. 2019; Brandon et al. 2009; Tichelman et al. 2019).

Since yawn contagion has been found to vary across the day (Giganti and Zilli 2011), we checked whether our yawning response sampling could be biased by the time periods during which the data were collected, depending on the availability of the study subjects. In neither setting did we find a significant effect (Tables 1 and 3), probably because the majority of the data was collected in the morning and in the afternoon (with little data collected at the very extremes of the day).

The use of a twofold approach, involving both experimental and naturalistic data collection, allowed the verification of the possible effect of different variables on yawn contagion. The results of the experimental trials show that the yawning response was significantly higher in the yawning than in the control video condition (Table 1; Fig. 2). This finding confirms that yawn contagion was present in the cohort of human subjects considered in this study (nulliparous and pregnant women) since it has been found in other segments of the population (Arnott et al. 2009; Provine 1989, 2005).

Yawn contagion may be affected by selective, top-down attentional biases (Massen and Gallup 2017), in addition to bottom-up, stimulus-driven attention (Attentional Bias Hypothesis, ABH; Palagi et al. 2020). Therefore, in the experimental setting we checked for selective attention to the stimulus and we found no significant influence of the time of attention to the stimulus source (video screen) on yawning (Table 1), which was high overall in both yawning and control video conditions, as well as in pregnant and nulliparous women. This finding reduces the probability that in our sample a selective attention bias may have accounted for the differences between stimulus (yawning/control) and reproductive status (pregnant/nulliparous) conditions. This is line with evidence indicating, directly or indirectly, that contagious yawning in humans may depend on bottom-up more than top-down selective attention (Norscia et al. 2020; for a review see Palagi et al. 2020). Age is another variable known to possibly affect yawn contagion rates (Bartholomew and Cirulli 2014). In our case, in the experimental setting there was a nonsignificant trend of the influence of age in the yawning response, possibly because the women under study fell within the relatively short reproductive age.

In the naturalistic setting we could verify the effect of a social bond between the trigger and the potential responder on the yawning response. Although the bond was restricted to two categories (strangers and acquaintances) owing to data constraints, and despite showing an inverse correlation with reproductive status, the bond had a significant effect on yawn contagion, which was more likely between subjects who knew each other than between strangers. This finding is in agreement with previous literature showing that relationship quality has an influence on yawn contagion, whose likelihood increases as the strength of the social bond increases (from strangers to acquaintances, friends, and lastly to family members; Norscia and Palagi 2011; Norscia et al. 2016). Norscia et al., (2020) found no difference between strangers and acquaintances when the yawns were heard but not seen, although friends and family responded at significantly higher rates than did those in the other categories. In the absence of the visual cue, it is probably more difficult for the potential responders to discern between subjects with whom they have reduced or no familiarity.

Importantly, our results from the experimental trials show that reproductive status (pregnant/nulliparous) had a significant effect on the yawning response in the yawning video condition but not in the control video condition (cf. Tables 2 and 3). Therefore, only yawning resulting from contagion—and not spontaneous yawning—was affected by pregnancy in our sample. Historical accounts report an increase of spontaneous yawning in the case of certain diseases (e.g., puerperal fever or hemorrhage; Walusinski 2010), and excessive yawning has indeed been indicated as a possible marker of disease in humans (Thompson and Simonsen 2015). Progesterone increases daytime drowsiness and sleeping time (Won 2015) and so it may increase spontaneous yawning rate during pregnancy. In this respect, we cannot exclude that the yawning stimulus might have preferentially primed the yawning motor response in pregnant women also because they experienced increased fatigue (despite showing similar levels of sleep to those of nulliparous women). An investigation on how spontaneous rates vary within subjects across pregnancy, possibly in relation to fatigue and tiredness, and how contagious yawning varies depending on the stimulus (e.g., babies/adults)—with hormonal and neurobiological correlates—could better clarify the above issues.

Overall, the different yawning response of pregnant women relative to women with no children can fall within the broad range of the behavioral changes that start occurring during pregnancy, such as motor activity and dietary choice variations (Crozier et al. 2009; Gradmark et al. 2011). Compared with childless women, pregnant women show increased sensitivity to emotional signals and facial expressions. For example, pregnant women were found to perceive infant cries in more differentiated ways than women with no offspring (Bleichfeld and Moely 1984; Yoshiaki 1985). As gestation progresses, pregnant women also show enhanced ability to encode and process emotional faces, especially related to distress (an emotional state; Keltner et al. 2019) as an evolutionary adaptation to motherhood, which requires hypersensitivity to emotional threat signals and contagion (Osório et al. 2018; Pearson et al. 2009). Our results fit with this scenario because they indicate enhanced responsiveness of pregnant women to yawning, which has been linked (with various degrees of evidence) to anxiety and distress in human and nonhuman primates (from lemurs to apes: e.g., Baker and Aureli 1997; Coleman and Pierre 2014; Leone et al. 2014; Palagi et al. 2019; Thompson 2014, 2017; Thompson and Bishop 2012; Zannella et al. 2015). Thompson (2014) has posited that cortisol (involved in the stress response) may be involved in yawn contagion, at least under certain situations. Another hypothesis, not mutually exclusive to the cortisol hypothesis, may be that yawn contagion is, to a certain extent, under the influence of oxytocin, considering that enhanced emotional recognition is one of the effects of oxytocin, whose levels largely increase during pregnancy (Domes et al. 2007; Preston 2013). In particular, oxytocin appears to increase the accuracy of the recognition of faces displaying angry and happy emotions, especially in women (Yue et al. 2018). Mariscal et al., (2019) found that yawn contagion in autism spectrum disorder (ASD) children was positively related to the blood concentration of oxytocin. The possible relationship between oxytocin and yawn contagion is supported by evidence that yawn contagion in humans follows the empathic gradient (sensu Preston and de Waal 2002), being highest between closely bonded subjects (Norscia and Palagi 2011; Norscia et al. 2020). Some features typical of mother-infant attachment, such as social recognition, bonding, and affiliation, are maintained in adulthood and promoted by oxytocin, which has been found to increase trust (Kosfeld et al. 2005), generosity (Zak et al. 2007), altruism (de Dreu et al. 2010), and both cognitive and affective empathy (Rodrigues et al. 2009; Shamay-Tsoory et al. 2013; Smith et al. 2014; Uzefovsky et al. 2015). One of the future steps is to evaluate the possible covariation between oxytocin and yawn contagion in both pregnant and nulliparous women. Beyond incorporating hormones, further studies could involve postmenopausal versus pregnant women and check how mothers react when they see their own fetus yawning on the echograph screen.

The possible connection between yawn contagion and increased social and emotional bonding is also suggested by the fact that some of the areas that seem to be involved in yawn contagion (such as the ventromedial-prefrontal cortex, superior temporal sulcus, amygdala, insula, posterior cingulate, and precuneus; Nahab et al. 2009; Platek et al. 2005; Schürmann et al. 2005) are also involved in mother-infant care, in mother’s enhanced sensitivity to the baby, and maternal brain changes occurring during pregnancy (Barba-Müller et al. 2019; Hoekzema et al. 2017; Kikuchi and Noriuchi 2015; Preston 2013; Rifkin-Graboi et al. 2015).

In summary, by showing increased occurrence of yawn contagion in pregnant women—a cohort of subjects that is specifically “programmed” to recognize and respond to others’ emotions—this study provides support for the hypothesis that yawn contagion may, at least under certain circumstances, underlie emotional contagion (EBH; Palagi et al. 2020). This process is considered by some scholars a basic form of empathy and occurs when an emotion is transferred from one individual to another, possibly via a motor perception–action mechanism, involving the matching of facial expressions and the resonance of the emotions that underlie such expressions (de Waal and Preston 2017).

The perception–action and the offspring care model both predict that subjects can preferentially attend the stimuli coming from closely bonded others, particularly caregiving individuals such as pregnant women toward babies (Preston 2013; Preston and de Waal 2002). Visual, top-down attention has limited effect on yawn contagion and does not follow a consistent familiarity trend in hominines because other factors, such as dominance, can come into play (Lewis et al. 2021; Norscia et al. 2020; Palagi et al. 2020). Hence, a possible bonding hypothesis between EBH and ABH is that yawn contagion can be influenced by emotional bonding and attention, mainly directed through bottom-up mechanisms.

Importantly, not all contagious yawning is triggered by emotional resonance, and that is not the point in question here. Contagious yawning also occurs between strangers (Norscia and Palagi 2011), and some people are consistently not susceptible to others’ yawns (Bartholomew and Cirulli 2014; Platek et al. 2003; Provine 1986, 1989). Contagious yawning is a form of yawning and—as such—can be related to nonemotional, individual and/or environmental factors, such as time of the day (Giganti and Zilli 2011), age (Bartholomew and Cirulli 2014), and possibly temperature (Gallup and Eldakar 2011). The perception–action mechanism itself is based on a theory in motor control that assumes that our physical motor acts are primed in the brain by observation of those in others, even if they do not bear emotional cues (Preston and de Waal 2002). Thus, contagious yawning can also be a nonemotional motoric response. The pivot around which this study revolves is the possible mechanism leading to the social variations observed in the occurrence of contagious yawning. Although still under debate (Adriaense et al. 2020; Massen and Gallup 2017), various physiological, neuroethological, and psychological studies sustain the possible connection between the social asymmetry of yawn contagion and emotional bonding. Some of the brain areas that appear to be involved in yawn contagion (Nahab et al. 2009; Platek et al. 2005; Schürmann et al. 2005) seem to overlap with those involved in emotional processing of internal and external stimuli and empathy (Palagi et al. 2020) and—importantly—with the maternal brain (Barba-Müller et al. 2019; Hoekzema et al. 2017; Kikuchi and Noriuchi 2015; Rifkin-Graboi et al. 2015). Highest levels of yawn contagion are associated with increased oxytocin levels (i.e., ASD children; Mariscal et al. 2019), enhanced social bonding (i.e., between friends and family; Norscia and Palagi 2011), and maternal prenatal bonding (i.e., in pregnant women; present study). Lower yawn contagion rates in association with levels of autistic traits were found to be related to attentive rather than background emotional empathy deficits (Helt et al. 2021). Finally, another study found that subjects who yawned in response to observing others’ yawns exhibited significantly higher empathy scores (Franzen et al. 2018).

Hence, although we cannot discard the possibility that other priming and motor mechanisms may also underlie the social asymmetry of yawn contagion, the hypothesis that this behavior has been coopted during evolution for emotional contagion still stands and gains further support.

## Data Availability

The raw data associated with this research are available in a Google Drive folder: https://drive.google.com/drive/folders/1O4MFD_nn-He0wtwyjozoi7JxxuP-WB8X?usp=sharing
